# ‘Molecular and Cellular Neuroscience’: Impacts of Eight Highly Cited Articles Published in This Section of *Brain Sciences* in 2024

**DOI:** 10.3390/brainsci16020188

**Published:** 2026-02-04

**Authors:** Swapan K. Ray

**Affiliations:** Department of Pathology, Microbiology, and Immunology, University of South Carolina School of Medicine, 6439 Garners Ferry Road, Columbia, SC 29209, USA; swapan.ray@uscmed.sc.edu; Tel.: +1-803-216-3420; Fax: +1-803-216-3428

**Keywords:** Alzheimer’s disease (AD), glioblastoma multiforme (GBM), amyotrophic lateral sclerosis (ALS), cognitive deficiency, cerebrovascular disease, Rett syndrome (RTT)

## Abstract

This year, the selection criteria for highly cited articles in the ‘Molecular and Cellular Neuroscience’ section of *Brain Sciences* were focused on publications that achieved a citation count of 10 or more during 2024. Applying this metric, the Editorial Office, in collaboration with myself as Associate Editor of the ‘Molecular and Cellular Neuroscience’ section of the journal, identified eight articles that not only exemplified the mission of this section but also made significant scientific contributions by advancing our current understanding of the molecular and cellular mechanisms underlying major and rare neurological disorders. These articles encompass miscellaneous topics, including Alzheimer’s disease (AD), chronic alcoholism, glioblastoma multiforme (GBM), amyotrophic lateral sclerosis (ALS), cognitive impairment, cerebrovascular disease, and Rett syndrome (RTT). Importantly, several contributions highlight experimental therapeutic strategies aimed at mitigating pathogenic mechanisms, offering promising avenues for translational research and future clinical applications.

## 1. Introduction

The ‘Molecular and Cellular Neuroscience’ section of *Brain Sciences* showcases original research and critical reviews that advance our understanding of the molecular and cellular mechanisms underlying neurological disorders. Our scope spans genetic analyses in human populations, in vitro systems, and in vivo models of major diseases such as Alzheimer’s disease (AD), glioblastoma multiforme (GBM or simply glioblastoma, the brain’s most malignant tumor), Parkinson’s disease (PD), amyotrophic lateral sclerosis (ALS), and multiple sclerosis (MS) and traumatic injuries in the central nervous system (CNS). We also consider new mechanistic studies in rare diseases in the CNS, such as Rett syndrome (RTT). We prioritize publishing studies that aim to elucidate novel molecular and cellular processes governing neural development, neuroplasticity, neuroinflammation, neurodegeneration, and functional neuroprotection.

Key areas include synaptic maintenance and remodeling [1], neuron–glia communication and extracellular vesicle signaling [2], epigenetic regulation of neuronal autophagy, modulating presynaptic function and synaptic remodeling with implications for neuroinflammation and plasticity [3], axonal regeneration and intrinsic growth programs [4], and neurodegenerative mechanisms with neuroregenerative strategies addressing repair in the CNS [5]. We also welcome investigations into signal transduction pathways, synaptic plasticity, various cell death mechanisms relevant to neurodegenerative conditions [6], and the gut–brain axis in neurodegenerative diseases with therapeutic implications [7,8].

Of particular interest are studies employing animal models with translational fidelity and bedside-to-bench approaches validating molecular signatures derived from human patients. Through this integrative framework, the ‘Molecular and Cellular Neuroscience’ section of *Brain Sciences* is designed to bridge fundamental molecular and cellular insights and clinical applications, fostering innovation in diagnostics and therapeutics for major and rare neurological diseases and injuries.

## 2. Impacts of the Following Eight Articles in the Field of Molecular and Cellular Neuroscience

### 2.1. Protein Kinase C (PKC) in Neurological Health: Implications for Alzheimer’s Disease and Chronic Alcohol Consumption

PKC represents a critical family of enzymes that orchestrate diverse cell signaling pathways underlying normal physiological function. Dysregulation of PKC has been implicated in major human diseases, including cancer, cardiovascular abnormalities, and neurological disorders, highlighting its biomedical importance [9]. Within the central nervous system, PKC is essential for synaptic plasticity, neuronal growth, learning, and memory, setting it as a key regulator of cognitive health [10]. Growing evidence underlines the contribution of altered PKC signaling to AD, where disrupted PKC isoform activity is linked to amyloid-β accumulation, impaired synaptic transmission, and progressive cognitive decline [11]. Additionally, chronic alcohol exposure modulates PKC-dependent pathways that drive maladaptive neural plasticity, underpinning addictive behaviors and dependence [12]. By examining the central roles of PKC in both AD pathogenesis and alcohol-related neuroadaptations, this review emphasizes the significance of PKC as a molecular meeting point for neurodegenerative and addictive mechanisms. This and other studies show that unravelling these interconnected pathways may guide the development of targeted therapeutics aimed at restoring PKC balance, ultimately offering new avenues for mitigating neurodegeneration, improving cognitive outcomes, and reducing alcohol-induced neural injury.

### 2.2. The Interplay Between Glioblastoma Cells and the Tumor Microenvironment: New Perspectives for Early Diagnosis and Targeted Cancer Therapy

GBM remains the prevalent and most aggressive primary brain tumor, with extreme heterogeneity and poor survival despite multimodal therapy. Persistent challenges stem from its complex tumor microenvironment (TME), including interactions among astrocytes, myeloid cells, and neoplastic cells, which drive relapse and therapeutic resistance. Recent studies have emphasized the pivotal role of extracellular vesicles (EVs) in mediating intercellular communication, promoting tumor progression, and contributing to an immunosuppressive TME [13]. EVs also carry molecular signatures that reflect GBM heterogeneity, making them promising tools for early diagnosis and disease monitoring [14]. Furthermore, plasma-derived EV profiling reveals distinct patient-specific biomarker patterns, underscoring their value in precision oncology [15]. By integrating literature-based evidence with bioinformatic analyses, this study highlights key astroglial and myeloid signaling pathways and positions EV-mediated communication as a crucial frontier for targeted GBM therapy and early diagnostic biomarker discovery.

### 2.3. The Diverse Roles of Reactive Astrocytes in the Pathogenesis of Amyotrophic Lateral Sclerosis

Reactive astrocytes undergo profound morphological, molecular, and functional remodeling in response to pathological stimuli, resulting in both neurotoxic and neuroprotective outcomes. Emerging evidence demonstrates that these astrocytic phenotypes are central to amyotrophic lateral sclerosis (ALS) pathogenesis, contributing to Ca^2+^ dysregulation, mitochondrial impairment, altered lipid and lactate metabolism, and glutamate excitotoxicity [16]. Recent studies have further highlighted astrocyte-driven neuroinflammation, oxidative stress, and metabolic dysfunction as key drivers of motor neuron degeneration in ALS. Additional research shows that astrocytic alterations exacerbate mitochondrial damage and inflammatory signaling, amplifying disease progression [17]. Broader neurodegeneration-focused analyses also position reactive astrocytes as dynamic contributors whose heterogeneity influences disease outcomes across neurodisorders, including ALS. Furthermore, comprehensive ALS studies underscore persistent challenges and the need for astrocyte-targeted therapies to address mechanisms such as excitotoxicity and mitochondrial dysfunction [18]. The insights from this article reinforce the therapeutic potential of targeting reactive astrocyte pathways to mitigate ALS progression.

### 2.4. Hippocampal Lactate Infusion Enhances Spatial Memory Correlated with Monocarboxylate Transporter 2 and Lactylation

This article provides compelling evidence that hippocampal lactate functions as more than a metabolic substrate, acting instead as a multifaceted regulator of neural plasticity and memory. By demonstrating that exogenous hippocampal lactate enhances spatial memory and upregulates key synaptic proteins, the findings support the emerging literature identifying lactate as an epigenetic modulator in the brain. Lactate-induced protein lactylation, observed here and increasingly recognized in other recent studies, suggests a direct link between metabolic state and transcriptional regulation, offering new insights into mechanisms underlying cognition [19,20]. The critical involvement of monocarboxylate transporter 2 (MCT2) highlights the importance of the process of neuron-specific lactate transport in mediating these effects, aligning with growing evidence of lactate-dependent signaling pathways in neurological health and disease [21]. Collectively, current reports and other results are expanding our understanding of the role of hippocampal lactate in brain function with a crucial function of MCT2 in these processes and underscore its therapeutic potential for cognitive disorders [22].

### 2.5. Indole-3-Carbinol and Its Derivatives as Neuroprotective Modulators

Signaling between brain-derived neurotrophic factor (BDNF) and its downstream tropomyosin receptor kinase B (TrkB) represents a central regulatory axis in neuronal survival, plasticity, and antidepressant responsiveness, and its impairment is strongly associated with neurodegenerative and psychiatric disorders [23]. Emerging evidence highlights the interplay between TrkB activation and the nuclear factor erythroid 2-related factor 2 (Nrf2)-antioxidant responsive element (ARE) antioxidant system, a critical defense pathway against oxidative stress-induced neuronal damage. Activation of the TrkB/phosphoinositide 3-kinase (PI3K)/protein kinase B (Akt) cascade enhances Nrf2 nuclear translocation, thereby strengthening endogenous neuroprotection. However, neurotrophin deficiency commonly observed in brain pathologies downregulates TrkB signaling, underscoring the therapeutic importance of restoring this pathway. Phytochemicals such as indole-3-carbinol (I3C) and diindolylmethane (DIM) mimic BDNF activity, promote Akt phosphorylation, and activate Nrf2 by disrupting the Keap1 complex, offering a dual mechanism to bolster neuronal resilience. Recent studies also reinforce the broader contributions of BDNF/TrkB signaling to neuroprotection, synaptic regulation, and transcriptional control in neurodegenerative disease contexts [24]. Together, this and other complimentary findings position I3C derivatives as promising candidates for multimodal neuroprotective therapy [25].

### 2.6. The Cerebrovascular Side of Plasticity: Microvascular Architecture Across Health and Neurodegenerative and Vascular Diseases

The extensive 600 km capillary network of the brain ensures precise nutrient delivery and maintains homeostasis through the blood–brain barrier (BBB), whose stability is upheld by low-turnover brain microvascular endothelial cells (BMECs). However, cerebrovascular plasticity allows this system to adapt to developmental, environmental, and pathological challenges [26]. Structural remodeling—triggered by hypoxia, aging, injury, and neurodegeneration—alters capillary segments, BMEC proliferation, and neurovascular coupling, directly influencing neuronal function and cognition [27]. Rapid shifts in cerebral blood flow occur within seconds in response to neural activity, while longer-term vascular restructuring unfolds over weeks to years depending on persistent stimuli, aging, or disease progression. Neurodegenerative conditions further reshape microvascular architecture, often through inflammation-driven mechanisms that impair vascular integrity and function. This and complementary reports highlight dynamic cerebrovascular responses to environmental perturbations, including angiogenesis, pruning, and endothelial cell turnover, underscoring the essential role of vasculature of the brain in resilience and vulnerability across the lifespan.

### 2.7. Alzheimer’s Disease, Obesity, and Type 2 Diabetes: Focus on Common Neuroglial Dysfunctions (Critical Review and New Data on Human Brain and Models)

Obesity, type 2 diabetes (T2D), and Alzheimer’s disease (AD) share convergent pathogenic mechanisms—insulin resistance, hyperglycemia, oxidative stress, mitochondrial dysfunction, and chronic neuroinflammation—positioning neuroglial cells as central drivers of neurodegeneration across these disorders. Astroglia, oligodendroglia, and microglia exhibit early dysfunction in T2D-AD models, with astrocytosis and microgliosis preceding amyloid pathology, highlighting neuroglia as early biomarkers and therapeutic targets [28]. Recent evidence emphasizes glia-mediated metabolic dysregulation, inflammasome activation, and impaired neuroglial communication as shared pathways accelerating cognitive decline in T2D and AD. Additional studies show that hyperglycemia-induced oxidative stress, blood–brain barrier breakdown, and mitochondrial injury exacerbate neurotoxic cascades in T2D-driven AD, reinforcing the metabolic–neurodegenerative link. Moreover, glial regulation of excitotoxicity through pathways such as tryptophan-kynurenine metabolism further connects metabolic and neurodegenerative vulnerability [29]. Altogether, this report and many other findings underscore neuroglial dysfunction as a unifying mechanism and promising multimodal therapeutic avenue for T2D-AD comorbidity.

### 2.8. Rett Syndrome and the Role of MECP2: Signaling to Clinical Trials

RTT, which is caused in 95% of cases by methyl CpG binding protein 2 (MECP2) mutations, remains a severe neurodevelopmental condition with limited therapeutic options [30]. Recent advances highlight expanding knowledge of epigenetic regulatory role of MECP2 and its widespread impact on neuronal gene expression and circuit stability. Emerging mechanistic studies reveal that loss of MECP2 leads to rapid dysregulation of hundreds of genes even before clinical decline, underscoring the urgency of early intervention strategies [31]. Parallel progress in gene therapy—particularly optimized adeno-associated virus-based MECP2 delivery systems—shows promise in improving survival, motor function, and neuronal signaling fidelity in preclinical models. Clinical translation is accelerating, with Taysha Gene Therapies-102 (TSHA-102) entering pivotal trials and demonstrating disease-modifying potential in early human studies. Overall, this article and other advances mark a transformative period in RTT research, bridging molecular insights and therapeutic innovation and offering renewed hope for targeted MECP2-based treatments [32].

## 3. Conclusions

The highly cited eight articles featured in the ‘Molecular and Cellular Neuroscience’ section of *Brain Sciences* in 2024 address critical aspects of neoplastic and neurodegenerative disorders within the CNS. These conditions, along with traumatic CNS injuries, constitute an escalating global health burden, profoundly impacting morbidity, mortality, and quality of human life. Studies presented here provide essential insights into various cellular and molecular mechanisms in several taxing neurological disorders, offering a foundation for innovative therapeutic strategies and translational advances. By elucidating complex pathophysiological processes, these articles serve as catalysts for scientific progress to mitigate disease impact and enhance patient outcomes. In the spirit of open science, we invite readers to engage with these influential contributions, fostering interdisciplinary collaboration and advancing discoveries that transform bench side findings into bedside solutions. Continued exploration and interdisciplinary research remain imperative to address the pressing challenges posed by CNS pathologies worldwide.

## Data Availability

No new data were created or analyzed in this study. So, data sharing is not applicable to this article.
